# Protective Effects of Methotrexate against Proatherosclerotic Cytokines: A Review of the Evidence

**DOI:** 10.1155/2017/9632846

**Published:** 2017-12-21

**Authors:** Arduino A. Mangoni, Angelo Zinellu, Salvatore Sotgia, Ciriaco Carru, Matteo Piga, Gian Luca Erre

**Affiliations:** ^1^Department of Clinical Pharmacology, College of Medicine and Public Health, Flinders Medical Centre, Flinders University, Adelaide, SA, Australia; ^2^Department of Biomedical Sciences, University of Sassari, Sassari, Italy; ^3^Quality Control Unit, University Hospital of Sassari (AOUSS), Sassari, Italy; ^4^Rheumatology Unit, University Clinic and AOU of Cagliari, Cagliari, Italy; ^5^Rheumatology Unit, Department of Clinical and Experimental Medicine, University Hospital of Sassari (AOUSS), Sassari, Italy

## Abstract

There is good epidemiological evidence that patients with autoimmune rheumatic disease states, particularly rheumatoid arthritis, have an increased risk of cardiovascular morbidity and mortality when compared to the general population. The presence of a chronic systemic proinflammatory state in this patient group disrupts the structural and functional integrity of the endothelium and the arterial wall, favouring the onset and progression of atherosclerosis. A significant role in the detrimental effects of inflammation on endothelial function and vascular homeostasis is played by specific proatherosclerotic cytokines such as tumour necrosis factor-alpha (TNF-*α*), interleukin-1 (IL-1), and interleukin-6 (IL-6). Recent systematic reviews and meta-analyses have shown that treatment with methotrexate, a first-line disease-modifying antirheumatic drug (DMARD), is associated with a significant reduction in atherosclerosis-mediated cardiovascular events, such as myocardial infarction and stroke, and mortality, when compared to other DMARDs. This suggests that methotrexate might exert specific protective effects against vascular inflammation and atherosclerosis in the context of autoimmune rheumatic disease. This review discusses the available evidence regarding the potential antiatherosclerotic effects of methotrexate through the inhibition of TNF-*α*, IL-1, and IL-6 and provides suggestions for future experimental and human studies addressing this issue.

## 1. Introduction

Autoimmune rheumatic diseases such as rheumatoid arthritis (RA) are characterized by the presence of a chronic inflammatory state affecting the joints as well as a number of other organs and tissues [1]. The prevalence of RA ranges between 0.1 and 5%, depending on specific ethnic groups and geographic locations, and is higher in females than in males [1]. Patients with RA have an increased risk of death [2]. The increased mortality in this patient group, as well as in other autoimmune rheumatic conditions, is primarily due to a relatively high prevalence of cardiovascular disease and its clinical consequences, mainly acute atherosclerosis-related events such as myocardial infarction and stroke [3–5]. The risks of myocardial infarction and stroke are, respectively, 38% and 24% higher in RA patients when compared to the general population [4, 5]. This suggests that RA favours the onset of vascular damage and atherosclerosis either through conventional cardiovascular risk factors, such as diabetes, hypercholesterolaemia, or cigarette smoking, or through alternative mechanisms [6, 7]. One possible alternative mechanism is represented by the autoimmune- and inflammation-mediated disruption of the structural and/or functional integrity of the endothelium. This leads to significant alterations in vascular homeostasis, driven by an impairment of nitric oxide (NO) synthesis by endothelial NO synthase (eNOS), that include a reduced endothelium-dependent vasodilatation, an increased leukocyte and monocyte adhesion to, and deposition in, the arterial wall, an increased intima-media thickness and arterial stiffness, and a prothrombotic tendency [8–13]. Notably, these abnormalities have been reported both in animal models of autoimmune rheumatic disease and in patients with RA [8, 14–18]. The “inflammatory theory” of atherosclerosis highlights the key role of specific cytokines, particularly tumour necrosis factor-alpha (TNF-*α*), interleukin-1 (IL-1), and interleukin-6 (IL-6), in disrupting endothelial integrity and vascular homeostasis [19, 20]. Given the presence of a chronic inflammatory state, and therefore a sustained vascular insult, it is likely that the pathophysiological role of proatherosclerotic cytokines is further augmented in RA. This might explain the increased cardiovascular risk reported in this patient group [7].

Over the last few years, an increasing number of *in vitro* and *in vivo* studies have sought to identify pharmacological strategies targeting inflammatory pathways for cardiovascular disease prevention and management [20–22]. A number of therapeutic agents commonly prescribed to combat immune activation and inflammation in RA might also exert salutary effects on endothelial function and vascular homeostasis. Recent epidemiological evidence suggests that methotrexate, an established first-line disease-modifying antirheumatic drug (DMARD), might exert protective effects against cardiovascular disease. This review discusses the role of cytokines and inflammation in the pathophysiology of endothelial dysfunction and atherosclerosis, the pharmacology of methotrexate, the reduced cardiovascular risk associated with its use, and the available evidence regarding the effects of this DMARD on proatherosclerotic cytokines and endothelial function.

## 2. Cytokines, Endothelial Dysfunction, and Atherosclerosis

**Figure 1:**
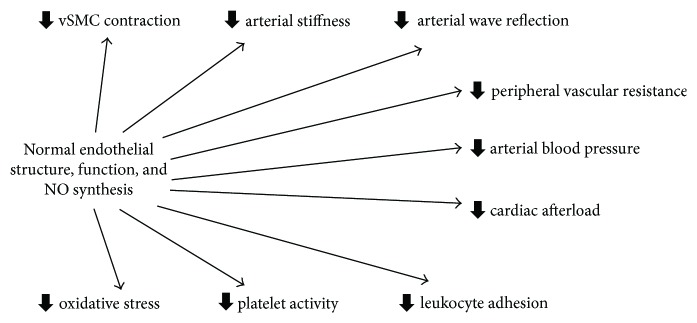
Endothelium, nitric oxide, and vascular homeostasis. NO: nitric oxide; VSMC: vascular smooth muscle cell.

The endothelium plays a key role in maintaining vascular homeostasis and protecting the vascular wall from a number of endogenous and exogenous proatherosclerotic insults [23]. A key role, in this context, is played by nitric oxide (NO), an endogenous messenger synthesised by the enzyme endothelial NO synthase (eNOS) [10]. NO regulates several important physiological processes, including vasodilation of arteries and arterioles, vascular tone, arterial stiffness, wave reflection, peripheral vascular resistance, blood pressure, and platelet function (Figure 1) [10]. A reduced NO synthesis by eNOS has been shown to be associated with virtually all cardiovascular risk factors [24]. Furthermore, clinical measures of endothelial dysfunction independently predict cardiovascular morbidity and mortality in several patient groups [25–27]. Therefore, the available evidence suggests that endothelial dysfunction is a key pathophysiological step in the sequence of events linking the presence of one or more cardiovascular risk factors with the development of atherosclerosis. Furthermore, endothelial dysfunction is useful in stratifying the risk of cardiovascular events at the population level and might represent an important target of therapies designed to mitigate such risk [28].

A significant number of cytokines, a group of low-molecular weight proteins, are produced in several cell types, such as endothelial cells, monocytes, and vascular smooth muscle cells, that play a key role in maintaining vascular homeostasis. Amongst them, the cytokines TNF-*α*, IL-1, and IL-6 have been extensively investigated not only from a pathophysiological point of view but also as therapeutic targets for novel cardioprotective therapies [21, 22, 29]. Both TNF-*α* and IL-1 are known to stimulate the synthesis of IL-6 during the process of immune activation and inflammation [30]. This, in turn, triggers several pathways that result in endothelial dysfunction, vascular inflammation and damage, and atherosclerosis [31]. The cellular effects of TNF-*α* and IL-1 are primarily mediated by the p38 mitogen-activated protein kinase (p38MAPK)/nuclear factor kappa-light-chain-enhancer of the activated B-cell (NF-*κ*B) pathways [32]. By contrast, the effects of IL-6 are mediated by the IL-6 receptor and the signal transducer protein gp130 [33].

Experimental studies have demonstrated the deleterious effects of TNF-*α*, IL-1, and IL-6 on endothelial function, vascular homeostasis, and cardiovascular risk and the protective effects of pharmacological agents targeting these cytokines on surrogate vascular markers (Table 1).

### 2.1. TNF-*α*

Treatment of endothelial cells with TNF-*α* has been shown to increase the expression of the inducible form of NO synthase (iNOS), decreasing at the same time the expression of the endothelial constitutive isoform eNOS [34]. While the maintenance of eNOS activity provides adequate NO synthesis for the regulation of several physiological and antatherosclerotic effects, an excessive NO synthesis by iNOS leads to the intracellular formation of reactive oxygen species with consequent development of endothelial dysfunction, apoptosis, and vascular damage [35]. Not surprisingly, several studies have also reported that TNF-*α* significantly impairs endothelium-dependent vasodilation, through an increase in reactive oxygen species, promotes the adhesion of leukocytes to the endothelium, and favours endothelial cell apoptosis [36–38]. Furthermore, TNF-*α* inhibits the activity of the enzyme dimethylarginine dimethylaminohydrolase, with consequent accumulation of the endogenous eNOS inhibitor asymmetric dimethylarginine (ADMA) [39, 40]. This effect is clinically relevant as higher plasma concentrations of ADMA have been shown to independently predict cardiovascular events in patients with a wide range of cardiovascular risk at baseline (Table 1) [41, 42].

Higher serum TNF-*α* concentrations have been associated with an increased risk of ischaemic stroke and recurrent coronary events in epidemiological studies [43, 44]. The key role of TNF-*α* in mediating the detrimental effects of inflammation on endothelial dysfunction and vascular homeostasis is also supported by the results of animal and human studies investigating the effects of specific TNF-*α* inhibitors. For example, treatment with adalimumab significantly reduced the adhesion of human leukocytes to endothelial cells and the expression of vascular cell adhesion molecule-1 (VCAM-1), intracellular adhesion molecule-1 (ICAM-1), and E-selectin [45]. Other studies have shown that treatment with adalimumab or etanercept improved clinical measures of endothelium-dependent vasodilation in patients with psoriasis, reduced the plasma concentrations of ADMA, and increased circulating endothelial progenitor cells in patients with RA (Table 1) [46–48].

### 2.2. IL-1

Increased production of IL-1 has been shown to induce leukocyte adhesion to the endothelium, exert procoagulant activity, and stimulate the growth and chemotaxis of vascular smooth muscle cells, key steps in the pathogenesis of atherosclerosis [49–52]. In animal studies, exposure to exogenous IL-6 causes an increase in coronary vasospastic responses to pharmacological challenge and intima thickening (Table 1) [53]. Moreover, multiple factors known to associate with atherosclerosis, such as cholesterol crystals, atheroprone oscillatory flow, hypoxia, and neutrophil extracellular traps, have recently been found to activate the critical IL-1beta producing NLRP3 inflammasome [22].

Polymorphisms in the IL-1 receptor antagonist (IL-1Ra) gene have been shown to have significant associations with the presence of single-vessel coronary artery disease, assessed by coronary angiography, in patients with ischaemic heart disease [54]. Conversely, pharmacological inhibition of IL-1 with anakinra reduces the concentrations of the potent endogenous vasoconstrictor endothelin-1 and arterial stiffness and improves clinical measures of endothelial function in patients with RA [55]. Similar effects of anakinra on endothelium-dependent vasodilation have been reported in animal models of diabetes (Table 1) [56]. In a recently completed randomized placebo-controlled trial in 10,061 participants with a previous myocardial infarction and C-reactive protein concentrations ≥2 mg/L, canakinumab, a monoclonal antibody targeting IL-1beta, significantly reduced the primary end-point of nonfatal myocardial infarction, nonfatal stroke, or cardiovascular death after a median follow-up of 3.7 years. Of the three subcutaneous doses of canakinumab studied (50 mg, 150 mg, and 300 mg every three months), only the 150 mg dose met the prespecified multiplicity adjusted threshold for statistical significance. Furthermore, there were no significant differences in all-cause mortality between treatment with canakinumab and placebo [57].

### 2.3. IL-6

IL-6 has been shown to upregulate the expression of the angiotensin II type-1 receptor in vascular smooth muscle cells, responsible for vasoconstriction, cell apoptosis, and proinflammatory effects, and to impair endothelium-dependent vasodilation in animal models [58]. Increased plasma IL-6 concentrations are significantly associated with a reduced endothelium-dependent vasodilation both in healthy subjects [59] and in patients with hypertension [60] or hypercholesterolaemia [61]. A significant association between plasma IL-6 concentrations and clinical markers of increased arterial stiffness has been reported in patients with hypertension (Table 1) [62]. A meta-analysis performed by the Emerging Risk Factors Collaboration has also shown that higher plasma IL-6 concentrations predict the risk of nonfatal myocardial infarction and coronary artery disease-related death, with a 25% increase in the risk of future vascular events for each increase in log IL-6 concentrations (RR 1.25, 95% CI 1.19 to 1.32) [63].

Furthermore, treatment with the IL-6 receptor inhibitor tocilizumab caused an increase in endothelium-dependent vasodilatation, and a concomitant reduction in arterial stiffness, in patients with RA [64, 65]. In another study, tocilizumab improved endothelial function and reduced markers of oxidative stress, inflammation, and thrombosis in patients with RA (Table 1) [66].

## 3. Methotrexate Pharmacology

**Figure 2:**
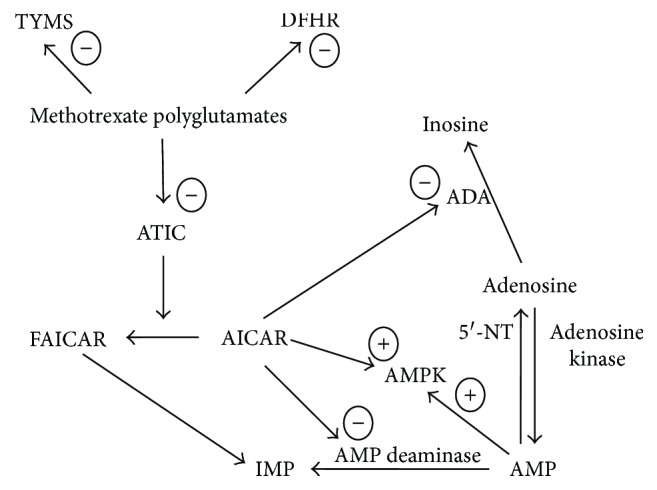
Intracellular effects of methotrexate. DHFR: dihydrofolate reductase; TYMS: thymidylate synthase; ATIC: aminoimidazole carboxamide ribonucleotide (AICAR) transformylase; FAICAR: 5-formamidoimidazole-4-carboxamide ribotide; IMP: inosine monophosphate; AMP: adenosine monophosphate; AMPK: 5′ adenosine monophosphate-activated protein kinase; ADA: adenosine deaminase; 5′-NT: 5′-nucleotidase; −: inhibition; +: activation.

Methotrexate, an analogue of the B vitamin folic acid, is a first-line synthetic DMARD for the management of RA and other autoimmune diseases that is normally administered once a week, either orally, subcutaneously, or intramuscularly, with doses ranging between 5 and 25 mg [67, 68]. Notably, methotrexate is the only DMARD that has demonstrated significant survival benefits in patients with RA [69–71]. After being transported into the cytoplasm, through the reduced folate carrier, methotrexate is converted into intracellular polyglutamates. The polyglutamate forms ensure the intracellular retention of methotrexate, allowing weekly administration despite the relatively short plasma elimination half-life (5–8 hours) [72]. The polyglutamates also mediate the immunomodulating and anti-inflammatory effects of methotrexate, by reducing the synthesis of purines, pyrimidines, and DNA through the inhibition of dihydrofolate reductase, thymidylate synthase, and aminoimidazole carboxamide ribonucleotide (AICAR) transformylase (ATIC, Figure 2) [73–75]. Furthermore, the inhibition of ATIC causes the accumulation of the substrate AICAR which, in turn, inhibits the enzymes adenosine deaminase and adenosine monophosphate (AMP) deaminase, involved in the catabolism of adenosine (Figure 2) [75]. Adenosine per se exerts significant anti-inflammatory effects, primarily through the A_2A_ and A_3_ receptors [76], and mediates the anti-inflammatory effects of methotrexate. In animal models of inflammation, methotrexate has been shown to increase AICAR and adenosine concentrations and to inhibit the accumulation of leukocytes, in exudates from carrageenan-inflamed air pouches. The administration of AMP deaminase partly reversed the methotrexate-mediated reduction in leukocyte accumulation. Additionally, the administration of 3,7-dimethyl-1-propargylxanthine, an A_2A_ receptor antagonist, but not 8-cyclopentyl-dipropylxanthine, an A_1_ receptor antagonist, suppressed the methotrexate-mediated reduction in leukocyte accumulation [77]. Furthermore, both AICAR and AMP activate the 5′ adenosine monophosphate-activated protein kinase (AMPK) [78]. AMPK activation is protective towards endothelial cell function and vascular homeostasis, by ensuring physiological NO synthesis, maintaining mitochondrial structure and function, and preventing oxidative stress and apoptosis [79]. Moreover, a role for AMPK in inhibiting vascular smooth muscle cell proliferation, a key proatherosclerotic event both in autoimmune rheumatic diseases and in the general population, has been proposed [80, 81]. There is experimental evidence that AMPK is a key mediator of the effects of methotrexate on inflammation and endothelial function. *In vitro* studies have shown that methotrexate, at concentrations between 0.1 and 0.5 *μ*M, increased AMPK phosphorylation and AMPK activity in perivascular adipose tissue cells. In these cells, the administration of palmitic acid, a proinflammatory agent, decreased AMPK phosphorylation; however, these effects were significantly reduced by pretreatment with either methotrexate or AICAR. Furthermore, methotrexate significantly reduced the phosphorylation of NF-*κ*B p65 and prevented the palmitic acid-mediated increase in the expression of TNF-*α* and IL-6, indicating anti-inflammatory effects. Silencing AMPK with AMPKa1/2-specific siRNA significantly decreased the inhibitory effects of methotrexate on palmitic acid-mediated NF-*κ*B p65 phosphorylation and on the expression of TNF-*α* and IL-6. Pretreatment with methotrexate prevented the impairment of acetylcholine-induced, endothelium-dependent vasodilation in rat aorta following palmitic acid administration. Notably, the protective effects of methotrexate on endothelial function were attenuated by cotreatment with compound C, an AMPK inhibitor [82].

## 4. Methotrexate and Cardiovascular Risk

Recent systematic reviews and meta-analyses have investigated the associations between methotrexate treatment and cardiovascular risk. Micha et al. identified observational studies in 66,334 patients with either RA (9 studies), psoriasis (1 study), or polyarthritis (1 study), that reported 6235 cardiovascular events. The median duration of follow-up in these studies was 5.8 years. Six studies compared methotrexate users versus never users, three compared current versus noncurrent users, and two compared initiators versus noninitiators. Cardiovascular endpoints included total or fatal cardiovascular disease (7 studies; two studies also provided separate estimates for myocardial infarction and ischaemic/haemorrhagic stroke), myocardial infarction (3 studies), and ischaemic stroke (1 study). Combined assessment of the included studies showed that methotrexate treatment was associated with a significant reduction in cardiovascular events (RR 0.79, 95% CI 0.73 to 0.87). Assessment of specific cardiovascular endpoints showed a similar effect size (overall cardiovascular disease events: RR 0.76, 95% CI 0.69 to 0.84; myocardial infarction: RR 0.82, 95% CI 0.71 to 0.96; stroke: RR 0.70, 95% CI 0.56 to 0.87) [83]. In a subsequent systematic review and meta-analysis, Roubille et al. identified 34 studies (28 studies in 236,525 RA patients, reporting 5410 cardiovascular events, and 6 studies in 220,209 patients with either psoriasis or psoriatic arthritis, reporting 2701 cardiovascular events). In studies conducted in RA patients, methotrexate use was associated with a reduced risk of all cardiovascular events (RR 0.72, 95% CI 0.57 to 0.91, *P* = 0.007) and myocardial infarction (RR 0.81, 95% CI 0.68 to 0.86, *P* = 0.01) when compared to other synthetic DMARDs. Although there was no significant effect of methotrexate on the risk of either stroke (RR 0.78, 95% CI 0.40 to 1.50) or major adverse cardiovascular events (RR 0.38, 95% CI 0.05 to 2.84), the number of studies assessing these endpoints was relatively low (one study for stroke and two studies for major adverse cardiovascular events, resp.) [84]. Therefore, the results of observational studies support the hypothesis that methotrexate exhibits specific protective cardiovascular effects when compared to other DMARDs.

## 5. Methotrexate and Proatherosclerotic Cytokines

In order to review the available evidence on the direct or indirect, mediated by adenosine, AICAR or AMPK activation, effects of methotrexate on proatherosclerotic cytokines, a PubMed literature search was conducted from inception to June 2017, using the following terms: methotrexate, adenosine, AICAR, AMPK, endothelium, inflammation, atherosclerosis, TNF-*α*, IL-1, and IL-6.

### 5.1. TNF-*α*

In *in vitro* studies, clinical concentrations of methotrexate (2 × 10^−8^ M and 2 × 10^−7^ M) have been shown to increase the release of soluble TNF receptor p75 from the cell surface [85]. This phenomenon is able to significantly inhibit the proinflammatory effects of TNF-*α* [86]. Adenosine is a known inhibitor of TNF-*α* expression through stimulation of the A_3_ receptor (Table 2) [87, 88]. There is recent evidence that methotrexate stimulates AMPK phosphorylation and activity, induces manganese superoxide dismutase mRNA and protein, and increases the expression of the cytoprotective genes haem oxygenase-1 and Bcl-2-related protein in human umbilical vein endothelial cells (HUVECs) and arterial endothelial cells (HAECs). The pretreatment of endothelial cells with TNF-*α* did not affect the methotrexate-mediated upregulation of manganese superoxide dismutase and haem oxygenase-1 mRNA [89]. Methotrexate (10^−8^ M to 10^−6^ M) and/or adenosine treatment has also been shown to prevent the TNF-*α*-induced (a) expression of ICAM-1 and VCAM-1 in human umbilical vein endothelial cells (HUVEC) [90] and (b) reduction of mitochondrial mass, membrane potential, and intracellular ATP concentrations, in endothelial cells through increased activity of eNOS [91]. Furthermore, adenosine has been shown to reduce the circulating concentrations of TNF-*α* and IL-6 in a mice model of sepsis (Table 2) [92]. By contrast, therapeutic concentrations of methotrexate, 0.1, 0.25, and 0.5 *μ*M, significantly increased the expression of the TNF-*α* receptor-associated factor 1 gene (~threefold change) and of the TNF-*α* receptor superfamily member 9 gene (~threefold change) in EA.hy 926 cells, derived from the fusion of primary endothelial cells with an epithelial tumour cell line [93].

### 5.2. IL-1

In an *in vitro* model of palmitate-induced endothelial dysfunction, AICAR-mediated AMPK activation has been shown to significantly reduce IL-1, IL-6, and VCAM-1 synthesis, by preventing the activation of the NLRP3 inflammasome [94]. Similarly, pharmacological activation of AMPK with oestradiol prevented the IL-1-induced expression of VCAM-1 and ICAM-1 in cultured human endothelial cells (Table 2) [95]. By contrast, therapeutic concentrations of methotrexate, 0.1, 0.25, and 0.5 *μ*M, significantly increased the expression of the IL-1 alpha gene (~threefold change) and of the IL-1 receptor-like 1 gene (~fourfold change) in EA.hy 926 cells [93].

### 5.3. IL-6

In db/db mice fed with Western diet, methotrexate treatment (4 mg/kg) caused a significant reduction in the circulating concentrations of IL-6 and TNF-*α*. These effects were associated with a significant increase in endothelium-dependent vasodilatation and a reduction in VCAM-1 [96]. In another study, methotrexate treatment (0.1–0.5 *μ*M) significantly reduced the expression of IL-6 and TNF-*α*. These effects were mediated by the activation of AMPK and were associated with an improvement in eNOS activity and endothelium-dependent vasodilation in rat aorta (Table 2) [82]. Adenosine has been shown to prevent the thrombin-mediated increased expression of IL-6, VCAM-1, ICAM-1, and E-selectin in HUVECs. These effects are mediated by the activation of the adenosine receptor A_2A_ [97]. Similarly, adenosine inhibited, in a dose-dependent fashion, the release of IL-6, VCAM-1, and ICAM-1 in HUVECs pretreated with either IL-1, TNF-*α*, or lipopolysaccharide (Table 2) [98].

## 6. Additional Vascular Effects of Adenosine, AICAR, and AMPK Activation

A number of studies have shown that adenosine, AICAR, and AMPK activation provide beneficial effects on endothelial function and vascular homeostasis that are independent of those on TNF-*α*, IL-1, or IL-6.

### 6.1. Adenosine Accumulation

There is good evidence that adenosine, through the activation of the A_2A_ and A_2B_ receptors, lowers blood pressure as a result of increased NO synthesis in the endothelium and direct vasodilation [99, 100]. Studies have also demonstrated the presence of central nervous system-mediated hypotensive effects through the activation of the A_3_ receptor [101]. Furthermore, the pharmacological activation of the A_2B_ receptor prevents the formation of atherosclerotic lesions and reduces the plasma concentrations of cholesterol and triglycerides, possibly through the reduced activation of the transcription factor sterol regulatory element-binding protein 1 in the liver [102, 103]. Activation of adenosine receptors seems also to modulate glucose homeostasis. However, the pathophysiological and clinical relevance of this finding is yet to be determined (Table 2) [104].

### 6.2. AICAR and Activation of 5′ Adenosine Monophosphate-Activated Protein Kinase (AMPK)

There is increasing evidence that AICAR and or/AMPK activation stimulates NO synthesis in the endothelium, enhances endothelium-dependent and endothelium-independent vasodilation, reduces blood pressure, prevents vessel restenosis, and increases cholesterol efflux capacity [105–110]. Furthermore, AMPK stimulates cellular glucose uptake, through GLUT-1 and GLUT-4 transporters, and glycolysis, through phosphorylation of two isoforms of the enzyme 6-phoshofructo-2-kinase: fructose-2,6-biphosphatase, with beneficial effects on glucose homeostasis [111–114]. There is also evidence that AMPK activation protects endothelial cells from the deleterious effects of chronic exposure to high concentrations of glucose and fatty acids and consequently reduces oxidative stress, inflammation, and endoplasmic reticulum stress (Table 2) [115, 116].

## 7. Discussion

The available evidence from systematic reviews and meta-analyses of observational studies in patients with either RA or other autoimmune rheumatic disease states suggests that the use of methotrexate is associated with a significant reduction in cardiovascular morbidity and mortality when compared to other DMARDs. Furthermore, a relatively small number of experimental studies have shown that methotrexate can exert beneficial effects on endothelial function and vascular homeostasis by preventing or blocking the effects of key proatherosclerotic cytokines such as TNF-*α*, IL-1, and IL-6. The reported effects are mediated either by methotrexate directly or through the activation of adenosine receptors, AICAR, or AMPK (Table 2). Pending further *in vitro* and *in vivo* studies investigating the exact mechanisms involved in such effects, a number of limitations need to be considered when interpreting the available data:

1. The dose of methotrexate used in some studies [96] and its consequent local concentrations in target cells and tissues are quite different from those normally observed in patients with autoimmune disorders.
2. No study has assessed the intracellular concentrations of methotrexate polyglutamates, as a factor mediating the effects of the drug on the study end-points.
3. The effects of methotrexate, adenosine, AICAR, or AMPK activation on proatherosclerotic cytokines were not compared to those of other synthetic or biologic DMARDs.
4. Other studies, not specifically investigating TNF-*α*, IL-1, and IL-6, have shown that methotrexate can also exert antiproliferative effects in human umbilical vein endothelial cells and EA.hy 926 cells [93, 117, 118] and reduce NO synthesis, possibly as a result of reduced availability of tetrahydrobiopterin, an essential cofactor for endothelial nitric oxide synthase, secondary to dihydrofolate reductase inhibition [119].
5. There is no direct evidence that the methotrexate-induced inhibition of TNF-*α*, IL-1, or IL-6 leads to sustained beneficial effects on endothelial function, atherosclerosis, arterial structure and function, and cardiovascular risk in human studies.

These issues should be accounted for in future studies investigating the effects of methotrexate on proatherosclerotic cytokines. In particular, the use of other DMARDs as comparator should help to determine whether methotrexate treatment exerts specific antiatherosclerotic effects that might help to explain its superiority, in terms of cardiovascular risk reduction, reported in observational studies. Furthermore, a comprehensive assessment of proatherosclerotic and antiatherosclerotic cytokines, such as transforming growth factor-*β*, IL-10, and IL-35 [120], as well as measures of endothelial cell proliferation, apoptosis, and nitric oxide synthesis, might provide additional mechanistic insights regarding the possible vasculoprotective effects of methotrexate and the potential rationale for combining methotrexate treatment with other interventional strategies targeting specific cytokines. In this context, the results of the Cardiovascular Inflammation Reduction Trial (CIRT, clinicaltrials.gov identifier NCT01594333), investigating the effects of methotrexate on myocardial infarction, stroke, and cardiovascular death in patients with type 2 diabetes or metabolic syndrome and stable coronary artery disease, will provide additional knowledge regarding the potential repurposing of methotrexate for cardiovascular risk management and prevention in different patient populations [121].

## 8. Conclusions

The evidence recently generated from experimental studies suggests that methotrexate, an anchor drug in the treatment of RA and other autoimmune disorders, might exert significant beneficial effects on endothelial function and vascular homeostasis by targeting key cytokines regulating the immune responses and inflammation pathways responsible for the development of atherosclerosis and thrombosis. However, further studies are required to fully establish the role of this DMARD as an antiatherosclerotic and cardioprotective agent.

## Figures and Tables

**Table 1:** Effects of the cytokines TNF-*α*, IL-1, and IL-6 on endothelial function and vascular homeostasis.

Cytokine	Reported effects
Tumour necrosis factor-alpha	Endothelial nitric oxide synthase activity ↓
	Inducible nitric oxide synthase activity ↑
	Reactive oxygen species ↑
	Endothelium-dependent vasodilation ↓
	Leukocyte adhesion ↑
	Endothelial cell apoptosis ↑
	Asymmetric dimethylarginine ↑

Interleukin-1	Endothelium-dependent vasodilation ↓
	Vasoconstrictor response to pharmacological challenge ↑
	Endothelin-1 ↑
	Leukocyte adhesion ↑
	Vascular smooth muscle cell growth ↑
	Intima thickness ↑
	Arterial stiffness ↑
	Coagulation ↑

Interleukin-6	Expression of angiotensin II type-1 receptor ↑
	Endothelium-dependent vasodilation ↓
	Arterial stiffness ↑
	Oxidative stress ↑
	Thrombosis ↑

↑: Increase; ↓: decrease.

**Table 2:** Effects of methotrexate, adenosine, AICAR, and AMPK activation on endothelial function and vascular homeostasis.

Mediator	Reported effects
Methotrexate	Release of soluble TNF-*α* receptor p75 ↑
	TNF-*α* expression/concentrations ↓
	IL-6 expression/concentrations ↓
	ICAM-1 expression ↓
	VCAM-1 expression ↓
	eNOS activity ↑
	Endothelium-dependent vasodilatation ↑
	Mitochondrial mass, membrane potential, and intracellular ATP concentrations ↑

Adenosine	TNF-*α* expression/concentrations ↓
	IL-6 expression/concentrations ↓
	ICAM-1 expression ↓
	VCAM-1 expression ↓
	E-selectin expression ↓
	eNOS activity ↑
	Blood pressure ↓
	Mitochondrial mass, membrane potential, and intracellular ATP concentrations ↑
	Formation of atherosclerotic lesions ↓
	Cholesterol concentrations ↓
	Triglyceride concentrations ↓

AICAR/AMPK	IL-1 expression/concentrations ↓
	IL-6 expression/concentrations ↓
	ICAM-1 expression ↓
	VCAM-1 expression ↓
	NO synthesis ↑
	Endothelium-dependent vasodilation ↑
	Endothelium-independent vasodilation ↑
	Blood pressure ↓
	Oxidative stress ↓
	Endoplasmic reticulum stress ↓
	Manganese superoxide dismutase induction ↑
	NF-*κ*B ↓
	Monocyte adhesion to endothelial cells ↓
	Restenosis ↓
	Cholesterol efflux capacity ↑
	Cellular glucose uptake ↑
	Glycolysis ↑

AICAR: aminoimidazole carboxamide ribonucleotide; AMPK: 5′ adenosine monophosphate-activated protein kinase; TNF-*α*: tumour necrosis factor-alpha; IL-1: interleukin-1; IL-6: interleukin-6; ICAM-1: intercellular adhesion molecule-1; VCAM-1: vascular cell adhesion molecule-1; ATP: adenosine triphosphate; eNOS: endothelial nitric oxide synthase; NF-*κ*B: nuclear factor kappa-light-chain-enhancer of the activated B-cell; NO: nitric oxide; ↑: increase; ↓: decrease.
